# Micronutrient Deficiency May Be Associated with the Onset of Chalkbrood Disease in Honey Bees

**DOI:** 10.3390/insects15040269

**Published:** 2024-04-12

**Authors:** Ratko Pavlović, Robert Brodschneider, Walter Goessler, Ljubiša Stanisavljević, Zoran Vujčić, Nenad M. Zarić

**Affiliations:** 1 Department of Biochemistry, Faculty of Chemistry, University of Belgrade, Studentski trg 12-16, 11000 Belgrade, Serbia; ratkopav@gmail.com (R.P.); zvujcic@chem.bg.ac.rs (Z.V.); 2 Department of Biology, University of Graz, Universitätsplatz 2, 8010 Graz, Austria; 3 Analytical Chemistry for Health and Environment, Institute of Chemistry, University of Graz, Universitätsplatz 1, 8010 Graz, Austria; walter.goessler@uni-graz.at; 4 Faculty of Biology, University of Belgrade, Studentski trg 12-16, 11000 Belgrade, Serbia; ljstanis@bio.bg.ac.rs

**Keywords:** *Apis mellifera*, *Ascosphaera apis*, element composition, nutrition, feeding

## Abstract

**Simple Summary:**

Chalkbrood is a fungal honey bee disease that effects honey bee larvae. It usually does not cause colony death. However, it can weaken the colony by reducing the number of bees and thus reducing chances for colony survival. It is known that poor nutrition can be behind the onset of honey bee diseases. Until now, the mineral content of larvae and mummies and the onset of chalkbrood disease have not been linked. Here, we show that there are differences in the elemental composition of larvae from colonies with different statuses of the chalkbrood disease. Mummies had higher concentrations of macroelements in comparison to apparently typically developed larvae from the same hive, while at the same time they had much lower concentrations of microelements that have known antifungal and antimicrobial activities. It may be that the lack of these elements contributes to the onset of chalkbrood disease.

**Abstract:**

Chalkbrood is a disease of honey bee brood caused by the fungal parasite *Ascosphaera apis*. Many factors such as genetics, temperature, humidity and nutrition influence the appearance of clinical symptoms. Poor nutrition impairs the immune system, which favors the manifestation of symptoms of many honey bee diseases. However, a direct link between dietary ingredients and the symptoms of chalkbrood disease has not yet been established. We show here that the elemental composition of chalkbrood mummies and healthy larvae from the same infected hives differ, as well as that mummies differ from larvae from healthy hives. Chalkbrood mummies had the highest concentration of macroelements such as Na, Mg, P, S, K and Ca and some microelements such as Rb and Sn, and at the same time the lowest concentration of B, As, Sr, Ag, Cd, Sb, Ba and Pb. Larvae from infected hives contained less Pb, Ba, Cs, Sb, Cd, Sr, As, Zn, Cu, Ni, Co, Mn, Cr, V and Al in contrast to healthy larvae from a disease-free apiary. This is the first study to demonstrate such differences, suggesting that an infection alters the larval nutrition or that nutrition is a predisposition for the outbreak of a chalkbrood infection. Though, based on results obtained from a case study, rather than from a controlled experiment, our findings stress the differences in elements of healthy versus diseased honey bee larvae.

## 1. Introduction

Honey bees, important pollinators, are threatened by the reduction of the availability and diversity of pollen and nectar sources due to land-use changes. Malnutrition can directly contribute to poor bee health, or indirectly effect their immunocompetence [1,2,3]. Honey bees consume macro- and micronutrients from nectar, pollen and water to adequately meet their nutritional requirements [4]. Feeding a diverse (multifloral) pollen diet may be a crucial factor for honey bee health [5,6,7,8]. However, the role of micronutrients in honey bee health is not yet completely understood [9]. Minerals and nutrients are vital for the reproduction and development of adult bees and larvae. It is known that some elements are essential to honey bees (Na, K, Ca, Mg, P), while others can be toxic (Al, Pb, Cd, Ba) [10,11]. Al is not a proven nutrient. It can replace other metals, especially magnesium, in protein-buried sites and induce conformational defects and changes in the protonation states of protein sidechains [12]. Pb can influence the activity of enzymes and antioxidants. It causes oxidative stress and mostly effects the nervous system [13]. Cd can have a catalytic role in the production of reactive oxygen species, increasing oxidative stress. It can interfere with DNA-repair mechanisms, ending in cell death [14]. Ba has a negative influence on K^+^ accumulation inside the cells, causing hyperkalemia, which causes the depolarization of membranes [13].

Colonies can be provided with additional essential minerals through supplemental feeding. However, the definite requirements and optimal amounts that honey bees need are still mostly unexplored [4,15]. Honey bee larvae are to a certain extend buffered against the changes in the food supply of the colony [16]. The effects of larval nutrition on the susceptibility of bees to disease have not been studied [17].

Chalkbrood is a honey bee brood disease caused by the fungus *Ascosphaera apis* (Ascomycota: Eurotiomycetes: Ascosphaerales). The disease is widespread worldwide, and there is evidence that the incidence of chalkbrood may be increasing [18,19,20,21]. The typical symptoms are irregular wax capping over the brood and uncapped cells scattered over the brood frames. Mummies can often be seen in cells, at the hive entrance or found on the bottom board, where they are removed by worker bees [22]. At first, dead larvae are covered by a fluffy white mold and swollen to the hexagonal shape of the cell. Later they shrink into ‘mummies’, and may become grey/black if spore cysts form [23]. Around 5–37% less honey is produced globally as a result of the disease, due to decreased productivity in affected bee colonies [24]. This happens due to a reduction in workforce caused by mycosis [25]. Although it is usually not lethal to the colony, it can hinder its development by reducing its population [26]. While adult bees are not susceptible to this pathogen, they can transmit the disease within and between beehives [19]. Inherited genetic traits, such as hygienic behavior, play a role in preventing the onset of chalkbrood disease [27]. Temperature and humidity are also factors that contribute to commencement of this disease [28,29]. The chilling of brood cells 24 h before or after they are sealed is an important predisposing factor for chalkbrood disease [30]. This fungus is best regarded as an opportunistic pathogen which is efficiently dispersed and very widespread, and its presence in the larvae does not necessarily cause the disease to appear; one or more predisposing conditions must occur at the same time for the disease to develop [23].

The development of healthy honey bee colonies is supported by adequate nutrition [4], but so far there has been no direct link between poor larval nutrition and chalkbrood disease in honey bees. As suggested by recent studies, negative effects of infectious viral and fungal diseases can be increased by the poor nutrition of honey bees [31]. At the same time, the nutritional physiology of honey bees can be negatively affected by common bee pathogens and parasites [32,33]. This holds the potential for deleterious feedback loops between poor nutrition and infectious disease, which may contribute to a spiral of deteriorating bee health [31]. Here, we investigated whether mummies and larvae from infected colonies show differences in elemental composition compared to larvae from healthy honey bee colonies.

## 2. Materials and Methods

Honey bee larvae and chalkbrood mummies were collected from two apiaries in Vršac, Serbia. One is positioned in an urban environment (45°06′35.3″ N 21°18′27.0″ E), and another one in a rural area (45°08′14.9″ N 21°20′04.2″ E), with around 3.7 km distance between them. The urban apiary hosted up to 15 hives, while the rural apiary had more than 60 hives with at least 100 more hives nearby. The urban apiary is considered disease-free, as the chalkbrood disease has not been present in the past ten years, while chalkbrood was occasionally present in the rural apiary. At the urban apiary, we sampled 25 larvae from each of three hives on 3 June 2023. From the rural apiary, we sampled 25 mummies and larvae from each of three hives which had moderate clinical symptoms of chalkbrood, and 25 larvae from each of three hives that did not show any symptoms of disease. Mummies were easy to spot on the frame, but only a few of them could be spotted on the bottom or landing board. Sampling was conducted during nectar dearth, and infected hives had notably smaller honey and pollen reserves in contrast to healthy hives. All sampled larvae were approximately 5 days old post-hatching and appeared healthy. They were collected from the comb cells closest to capped brood, and the first signs of the capping process were visible. Mummies were collected only from frames (not from the bottom board) only from those cells that were partially opened by bees, with no regard to their color. The samples were pooled, each containing 75 larvae or mummies, leaving one pooled sample, each of the following: (1) larvae from the urban disease-free apiary, (2) larvae from rural hives that do not show symptoms of chalkbrood disease, (3) larvae from rural hives that show symptoms of chalkbrood disease and (4) mummies from rural colonies with chalkbrood disease. Each of the pooled samples were analyzed in triplicate, adding up to *n* = 12.

### 2.1. Chemicals and Standards

A purification system (Milli-Q, Merck Millipore, Darmstadt, Germany) was used to provide purified water (18.2 MΩ cm). Nitric acid (HNO_3_) Rotipuran p. a. ≥ 65% (Carl Roth, Karlsruhe, Germany) was sub-boiled with an MLS duoPUR (MLS, Leutkirch, Germany) prior to its use for the preparation of samples. For internal standards and the preparation of calibration standards, we used ICP Single-Element Standards Certipur (Merck Millipore, Darmstadt, Germany) and Single Element Standards for ICP (Carl Roth, Karlsruhe, Germany). Fifteen and fifty mL Cellstar polypropylene tubes (Greiner Bio-One International GmbH, Kremsmünster, Austria) were used for the preparation of all solutions.

### 2.2. Sample Preparation

The sample preparation was adopted from [34]. In short, 100 mg of freeze-dried and homogenized honey bee samples was digested in an ultraCLAVE IV microwave digestion system (MLS GmbH, Leutkirch, Germany) using 5 mL of concentrated HNO_3_. Each digestion was accompanied by three digestion blanks (5 mL conc. HNO_3_) and three reference materials BOVM-1 “Bovine muscle powder” (NRC, Ottawa, ON, Canada). After digestion, samples were left to cool, transferred to 50 mL Cellstar tubes, and diluted with ultrapure water to a final volume of 50 mL (10% (*v*/*v*) nitric acid).

### 2.3. Determination of Element Concentrations

Element concentrations were determined as described [34] using inductively coupled plasma mass spectrometry—ICPMS (Agilent ICPMS 7700x, Waldbronn, Germany). We have used an external calibration curve with six points and four concentration ranges. The calibration curve was made in 10% HNO_3_ to match the sample matrix. Calibration curve ranges (Text S1), the performance of the instrument (Table S1), the selected mass, the tune mode and internal standard for correction for each element analyzed and the detection limits (Table S2) are reported in the Supplementary Material.

### 2.4. Quality Control

Quality control was achieved through the continuous addition of Be, Ge, In and Lu (200 μg L^−1^ in 1% *v*/*v* HNO_3_) and analyses of drift standards (after every 10 samples). Extraction efficiency was evaluated by subjecting BOVM-1: Bovine Muscle Certified Reference Material for Trace Metals and other Constituents (NRC, Canada) through the same digestion process as the samples (Table S3). In addition, accuracy was also evaluated using SRM 1643f Trace elements in natural water (National Institute of Standards & Technology, Gaithersburg, MD, USA) (Table S4).

### 2.5. Statistical Analyses

Microsoft Excel 2021, version 2108 and IBM SPSS Statistics 25 were used to process the data statistically. Descriptive statistics including mean concentrations and standard deviations were calculated (Table S5). To assess statistically significant differences between samples, an ANOVA followed by Tukey’s HSD test was applied to the dataset (Table S5).

## 3. Results and Discussion

**Figure 1:**
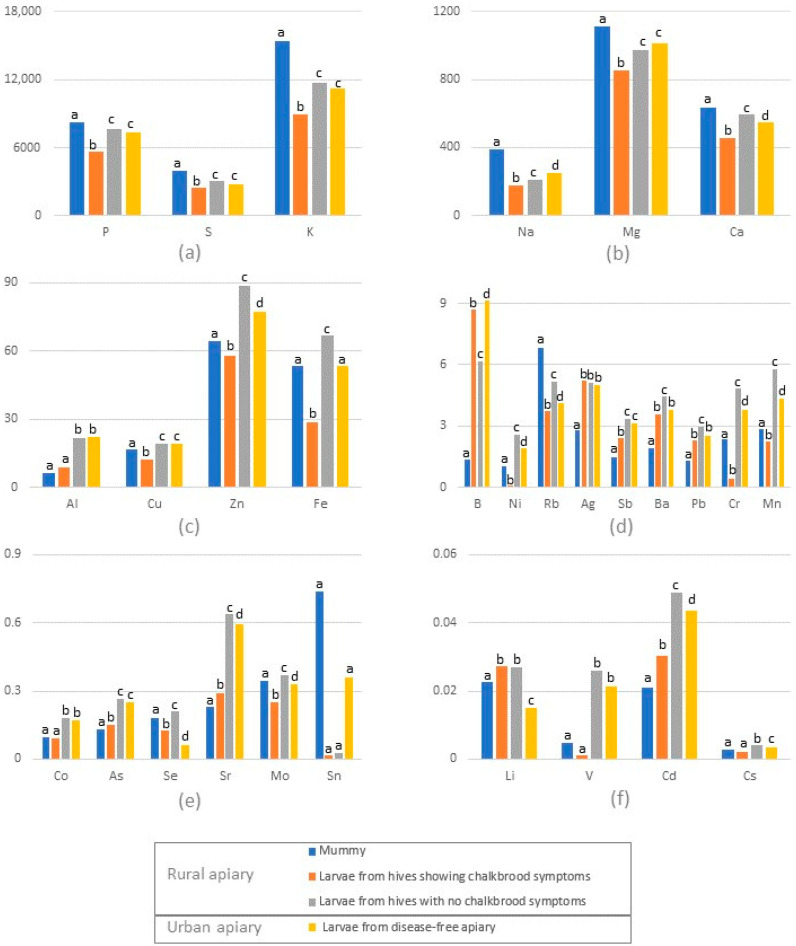
(**a**–**f**) Element concentrations (mg kg^−1^ dry weight) in mummies, larvae from hives showing chalkbrood symptoms and larvae from hives not showing chalkbrood symptoms from rural apiary and larvae from disease-free urban apiary (*n* = 12); different lower-case letters (a, b, c and d) represent statistically significant differences in element concentrations between samples.

Chalkbrood mummies had higher concentrations of macroelements such as Na, Mg, P, S, K, Ca and some microelements, like Rb, compared to all other samples (Figure 1a,b,d, Supplementary Material Table S5). At the same time, the mummies had the lowest concentrations of B, As, Sr, Ag, Cd, Sb, Ba and Pb (Figure 1d,e). Mummies and larvae from infected hives contained statistically less Al, Cr, Mn, Co, Ni, Cu, Zn, As, Sr, Cd, Sb and Cs compared to larvae from uninfected hives in the same apiary and to larvae from the disease-free apiary (Figure 1, Table S5).

This could indicate that the larvae from infected hives consumed a more uniform diet that is rich in macroelements, but lacking in microelements, compared to larvae from healthy hives, where the larvae are fed based on diverse food sources. Earlier studies concluded that the composition of the food is more important than the amount of food consumed [35]. It could be that honey bees from the infected hives either collected less pollen or the pollen collected was of a lower diversity. There are a number of reasons for this, including genetics (pollen-hoarding selection) [27,36], feedback between poor nutrition and infectious disease or intraspecific competition between honey bee colonies [31,37]. It is possible that the foragers from infected hives were outcompeted for pollen by honey bees from nearby, uninfected hives in the same apiary. Previous studies have shown that bees from different hives in the same apiary accumulate different amounts of elements in them. In the same study, bees from the same hive also had different element concentrations [34]. Recent studied concluded that most of the elements accumulated in bees originate from the food they eat [38,39,40]. This same food is processed and used to feed larvae; hence it is safe to assume that not all larvae receive the same elemental composition of food. Even in a normally functioning colony with adequate resources, some larvae are seemingly stochastically ignored by nurses for sufficient time to result in some amount of “hunger”, and the deprived larva pheromonally sends a “hunger signal” to positively influence its chances of being fed [41]. This means that not all larvae are fed equally, hence not receiving the same quantity and quality of food [42,43]. There is a possibility that the infected larvae try to compensate for the nutrient deficiency caused by the nutrient consumption of *Ascosphaera apis* in the same way in which infected bees try to compensate for the energetic stress caused by *Nosema ceranae*, through eating more, by sending more “hunger signal”, and in this way positively influences its chances to receive more jelly [41,44].

Based on the data presented here, we suggest that the deficiency of some elements could either be a possible cause contributing to the onset of chalkbrood disease or a consequence of the infection. Mainly, B, As, Sr, Ag, Cd, Sb, Ba and Pb are interesting elements, as we found their concentrations in mummies to be much lower than in larvae from the same colonies (Figure 1). As already mentioned, not all larvae from one colony will turn into mummies. Only the infected larvae that are mummified have lower concentrations of the mentioned elements. In addition, larvae and mummies from hives that show symptoms of chalkbrood had lower concentrations of Al, Cu, Zn, Ni, Cr, Mn, Co, Mo, V and Cs compared to larvae from hives that did not show the onset of disease and to the larvae from hives in the disease-free apiary. The fact that larvae contain more macroelements in healthy colonies in comparison to colonies with chalkbrood symptoms strengthens our hypothesis that chalkbrood is associated with poor nutrition. Larvae in healthy hives are generally better fed.

Boron concentration in the mummies is very low in contrast to the larvae from the infected hives, but also from all other hives. Several boron-containing compounds show excellent antifungal activity against important fungal pathogens *Mycosphaerella fijiensis* and *Colletotrichum sublineolum* [45]. Low B concentrations in mummies compared to larvae from the same hive suggests, as already mentioned, that not all larvae are fed food with same elemental composition. Aluminum and its salts have attracted attention in recent decades as an alternative to chemical antifungals. It could be referred to as an organic antifungal. They have been shown to be effective against pathogens from the *Ascomycota* phylum [46]. Al is mostly excreted by honey bees [47]. This could be the reason it is well tolerated and why Zaric et al. [48] found levels in live honey bees that are 10-20 times higher than in previous studies. Our results show a lower zinc content in larvae from infected hives and mummies compared to larvae from healthy colonies. Zinc supplementation increases the antioxidant defenses of honey bees [49]. A preference for zinc-treated food has been observed in *Apis mellifera*, underlining the importance of zinc for honey bee colonies [50]. Zinc oxide and silver nanoparticles inhibit *Ascosphaera apis* [51]. We observed a much lower silver concentration in mummies in contrast to larvae from infected but also from healthy colonies. Ag is well known to be antimicrobial and antibacterial [52,53]. It is possible that only larvae fed a low quantity of certain microelements are mummified while others are protected by these same elements. Larvae prior to mummification were probably fed a diet low in this element, lowering its antimicrobial activity and thus providing the opportunity for the onset of chalkbrood. In addition, most of the elements, including V, Cr, Mn and Sr, which we found in very low concentrations in mummies and larvae from infected colonies, are known for their antifungal properties [54,55,56]. Hence, it could be hypothesized that a lack of these elements favors the outbreak of chalkbrood; we suggest further controlled studies.

## 4. Conclusions

Chalkbrood is caused by *Ascosphaera apis* spores, which must be present in the colony before clinical symptoms break out. In an infected colony, some of the larvae develop into adult bees and some of them die and become mummies. Our data show that some elements have higher concentrations in mummies compared to healthy larvae from the same colony, while for other elements the opposite is true. This is possibly the outcome of the qualitative properties of the food that is fed to these larvae, which dependents on the pollen composition. Infected larvae that will turn into mummies are most likely fed a higher quantity of food that is rich in some elements (P, S, K, Na, Mg, Ca, Cu, Zn, Rb, Cr, Mn, and Mo), while lacking others (Al, B, Ag, Sb, Ba, Pb, As, Sr and Cd), some of which exhibit antifungal properties. The lack of antifungal elements in the larval diet could be the reason behind the onset of chalkbrood and the mumification of some larvae within infected hives. However, sampling more locations and different timepoints as well as different ages of larvae is needed for a more conclusive conclusion.

## Data Availability

The data are available in Supplementary Material Table S5.

## Supplementary Materials

The following supporting information can be downloaded at https://www.mdpi.com/article/10.3390/insects15040269/s1, Text S1: Calibration-curve ranges; Table S1. Performance of the ICPMS; Table S2. Selected mass, tune mode, internal standard and detection limits (LoD); Table S3. Certified and determined values for elements in NIST SRM 1643f Trace Elements in Natural Water; Table S4 Certified and determined values for elements in CRM BOVN-1 Bovine Muscle Powder; Table S5. Descriptive statistics and ANOVA (mg kg^−1^ dry weight ± standard deviation).
