# Supplementary material for: Micronutrient Deficiency May Be Associated with the Onset of Chalkbrood Disease in Honey Bees
Source: Insects. 2024 Apr 12;15(4):269. doi: 10.3390/insects15040269 (PMC11050715; doi:10.3390/insects15040269)
Supplement: Supplementary file 1 [file insects-15-00269-s001.zip › insects-2902443-supplementary.pdf]

Text S1:
Calibration curve ranges:
0.0100–5.00 µg L^{-1}: Li, V, Cr, Co, Ni, As, Se, Mo, Ag, Cd, Sn, Sb, Cs, Tl, Pb and U
0.1–50 µg L^{-1}: B, Ba, Cu, Rb, and Sr
1.00–500 µg L^{-1}: Al, Mn, Fe, Zn
100–50,000 µg L^{-1}: Na, K, Ca, Mg, P and S

Table S1. Performance of the ICPMS

Parameter	No-gas mode	Collision mode	Reaction mode
Cell gas	-	He	H_2
^{7}Li [CPS per µg L^{-1}]	$2.8*10^3$	-	-
^{59}Co [CPS per µg L^{-1}]	-	$2.6*10^3$	$4.5*10^2$
^{89}Y [CPS per µg L^{-1}]	$17*10^3$	$3.3*10^3$	$7.2*10^3$
^{205}Tl [CPS per µg L^{-1}]	$7.8*10^3$	$6.3*10^4$	$7.2*10^4$
average RSD [%]	2.5	2.5	3.5
$^{140}Ce^{16}O/^{140}Ce$ [%]	1.7	0.9	2.4
$^{140}Ce^{2+}/^{140}Ce^{+}$ [%]	2.5	0.7	0.3

Table S2. Selected mass, tune mode, internal standard and detection limits (LoD)

**Monitored isotope**	**Tune mode**	**Internal standard**	**Detection limit*** **($\mu g\ L^{-1}$)**
^{7}Li	No-gas	^{9}Be	0.0028
^{11}B	No-gas	^{9}Be	0.23
^{23}Na	He	^{9}Be	27
^{24}Mg	He	^{9}Be	11
^{27}Al	No-gas	^{9}Be	9.3
^{31}P	He	^{9}Be	27
^{32}S	He	^{9}Be	1641
^{39}K	He	^{9}Be	9.8
^{43}Ca	He	^{9}Be	154
^{51}V	He	^{74}Ge	0.02
^{53}Cr	He	^{74}Ge	0.08
^{55}Mn	He	^{74}Ge	0.09
^{56}Fe	He	^{74}Ge	2.7
^{59}Co	He	^{74}Ge	0.004
^{60}Ni	He	^{74}Ge	0.9
^{65}Cu	He	^{74}Ge	0.62
^{66}Zn	He	^{74}Ge	9.3
^{75}As	He	^{74}Ge	0.006
^{78}Se	H_2	^{74}Ge	0.01
^{85}Rb	He	^{74}Ge	0.023
^{88}Sr	He	^{74}Ge	0.1
^{98}Mo	No-gas	^{74}Ge	0.02
^{107}Ag	No-gas	^{115}In	0.003
^{111}Cd	No-gas	^{115}In	0.009
^{118}Sn	No-gas	^{115}In	0.05
^{121}Sb	No-gas	^{115}In	0.02
^{133}Cs	No-gas	^{115}In	0.002
^{137}Ba	No-gas	^{115}In	0.17
^{205}Tl	No-gas	^{175}Lu	0.0012
^{208}Pb	No-gas	^{175}Lu	0.03
^{238}U	No-gas	^{175}Lu	0.0003

*LoD = $mean_{blanks} + 3*\sigma_{blanks}$

Table S3. Certified and determined values for elements in NIST SRM 1643f Trace Elements in Natural Water

**Element**	**Cert. mass conc. [µg kg^{-1}]**			**Analyzed mass conc. [µg kg^{-1}] (n=3)**		
Li	16.42	±	0.35	15.93	±	0.16
B	150.8	±	6.6	140.5	±	1.4
Na	18640	±	240	23572	±	872
Mg	7380	±	58	7242	±	333
Al	132.5	±	1.2	139.8	±	2.2
K	1913.3	±	9.0	2041	±	51
Ca	29140	±	320	28440	±	170
V	35.71	±	0.27	32.32	±	0.71
Cr	18.32	±	0.10	17.12	±	0.38
Mn	36.77	±	0.58	36.9	±	1.2
Fe	92.51	±	0.77	90.7	±	2.2
Co	25.05	±	0.17	23.30	±	0.42
Ni	59.2	±	1.4	52.68	±	0.95
Cu	21.44	±	0.70	21.39	±	0.53
Zn	73.7	±	1.7	331.3	±	6.3
As	56.85	±	0.37	55.9	±	1.6
Se	11.583	±	0.078	10.82	±	0.41
Rb	12.51	±	0.12	11.94	±	0.31
Sr	311	±	18	183.2	±	6.2
Mo	114.2	±	1.7	109.26	±	0.76
Ag	0.9606	±	0.0053	1.320	±	0.026
Cd	5.83	±	0.13	7.17	±	0.12
Sb	54.90	±	0.39	54.36	±	0.38
Ba	513.1	±	7.3	467.2	±	4.7
Tl	6.823	±	0.034	6.715	±	0.060
Pb	18.303	±	0.081	19.52	±	0.23

Table S4 Certified and determined values for elements in CRM BOVN-1 Bovine Muscle Powder (*information values)

Element	Cert. mass conc. [mg kg^{-1}]			Analyzed mass conc. [mg kg^{-1}] (n=6)		
B	600	±	400	236	±	37
Na	2100	±	100	1779	±	14
Mg	960	±	95	875.1	±	76
Al*	1.7	±	/	0.82	±	0.38
P	8360	±	450	7055	±	51
S*	8000	±	/	6787	±	63
K	15200	±	400	13691	±	110
Ca	145	±	20	130.2	±	7.8
V*	0.005	±	/	0.0030	±	0.0013
Cr*	0.071	±	/	0.053	±	0.036
Mn	0.37	±	0.09	0.308	±	0.011
Fe	71.2	±	9.2	63.2	±	1.3
Co	0.007	±	0.003	0.00552	±	0.00022
Ni*	0.05	±	/	0	±	/
Cu	2.84	±	0.45	2.412	±	0.021
Zn	142	±	14	130.4	±	1.3
As	0.009	±	0.003	0.0085	±	0.0013
Se	0.076	±	0.010	0.0625	±	0.0040
Rb	28.7	±	3.5	24.23	±	0.35
Sr	0.052	±	0.015	0.0517	±	0.0052
Mo	0.08	±	0.06	0.0618	±	0.0017
Cd	0.013	±	0.011	0.01045	±	0.00098
Sb*	0.01	±	/	0.0038	±	0.0038
Cs*	0.05	±	/	0.03070	±	0.00042
Ba*	0.05	±	/	0.0161	±	0.0057
Pb	0.38	±	0.024	0.39	±	0.13

Table S5. Descriptive statistics and ANOVA (mg kg^{-1} dry weight ± standard deviation) (n=12)

	Rural apiary with signs of disease									Urban, Disease-free apiary			
Element	Mummy			Healthy Larvae from infected hive			Healthy Larvae from uninfected hive			Healthy Larvae from disease-free apiary			ANOVA
Li	0.02274^{a}	±	0.00017	0.0271^{b}	±	0.001	0.02687^{b}	±	0.00022	0.01518^{c}	±	0.00023	F(3,8)=328.504, p<0.0001
B	1.34^{a}	±	0.046	8.7^{b}	±	0.12	6.162^{c}	±	0.056	9.142^{d}	±	0.091	F(3,8)=5587.784, p<0.0001
Na	391.8^{a}	±	6.5	180.8^{b}	±	5.3	212.5^{c}	±	6.1	250.6^{d}	±	6.5	F(3,8)=692.143, p<0.0001
Mg	1113^{a}	±	21	852^{b}	±	21	972^{c}	±	29	1016^{c}	±	27	F(3,8)=59.013, p<0.0001
Al	6.21^{a}	±	0.2	8.89^{a}	±	0.99	21.86^{b}	±	0.97	22.1^{b}	±	1.9	F(3,8)=153.717, p<0.0001
P	8190^{a}	±	120	5620^{b}	±	130	7640^{c}	±	200	7360^{c}	±	190	F(3,8)=137.794, p<0.0001
S	3954^{a}	±	25	2510^{b}	±	110	3070^{c}	±	140	2812^{c}	±	99	F(3,8)=108.255, p<0.0001
K	15390^{a}	±	210	8950^{b}	±	190	11760^{c}	±	320	11190^{c}	±	300	F(3,8)=308.893, p<0.0001
Ca	638^{a}	±	16	455^{b}	±	12	592.2^{c}	±	8.4	548^{d}	±	11	F(3,8)=123.870, p<0.0001
V	0.0049^{a}	±	0.0022	0.0006^{a}	±	0.0011	0.02608^{b}	±	0.00057	0.0214^{b}	±	0.0036	F(3,8)=53.279, p=0.0001
Cr	2.353^{a}	±	0.086	0.444^{b}	±	0.046	4.83^{c}	±	0.1	3.81^{d}	±	0.52	F(3,8)=150.608, p<0.0001
Mn	2.867^{a}	±	0.033	2.243^{b}	±	0.034	5.789^{c}	±	0.074	4.324^{d}	±	0.054	F(3,8)=2819.921, p<0.0001
Fe	53.3^{a}	±	1.1	28.43^{b}	±	0.62	66.9^{c}	±	1.0	53.2^{a}	±	2.1	F(3,8)=424.605, p<0.0001
Co	0.09773^{a}	±	0.00086	0.093^{a}	±	0.0024	0.1807^{b}	±	0.0013	0.1722^{b}	±	0.0071	F(3,8)=449.773, p<0.0001
Ni	1.028^{a}	±	0.043	0.069^{b}	±	0.042	2.597^{c}	±	0.074	1.92^{d}	±	0.31	F(3,8)=135.016, p<0.0001
Cu	16.57^{a}	±	0.29	12.32^{b}	±	0.19	19.19^{c}	±	0.27	18.97^{c}	±	0.16	F(3,8)=557.596, p<0.0001
Zn	64.6^{a}	±	1.9	58.13^{b}	±	0.48	88.6^{c}	±	1.2	77.19^{d}	±	0.68	F(3,8)=397.611, p<0.0001
As	0.1332^{a}	±	0.0085	0.1521^{b}	±	0.0017	0.2635^{c}	±	0.0022	0.2505^{c}	±	0.0078	F(3,8)=379.237, p<0.0001
Se	0.183^{a}	±	0.0087	0.1253^{b}	±	0.0038	0.2121^{c}	±	0.0033	0.063^{d}	±	0.0019	F(3,8)=501.709, p<0.0001
Rb	6.85^{a}	±	0.075	3.75^{b}	±	0.047	5.175^{c}	±	0.052	4.12^{d}	±	0.048	F(3,8)=1806.510, P<0.0001
Sr	0.2297^{a}	±	0.0015	0.29^{b}	±	0.01	0.6361^{c}	±	0.0086	0.5954^{d}	±	0.0038	F(3,8)=2679.616, p<0.0001
Mo	0.3468^{a}	±	0.0064	0.2498^{b}	±	0.0045	0.3672^{c}	±	0.0036	0.3295^{d}	±	0.0032	F(3,8)=377.805, p<0.0001
Ag	2.786^{a}	±	0.029	5.211^{b}	±	0.07	5.11^{b}	±	0.11	5.024^{b}	±	0.062	F(3,8)=790.820, p<0.0001
Cd	0.02115^{a}	±	0.00097	0.03026^{b}	±	0.00025	0.0488^{c}	±	0.0015	0.04347^{d}	±	0.00092	F(3,8)=452.412, p<0.0001
Sn	0.7^{a}	±	1.2	0.0161^{a}	±	0.0037	0.02726^{a}	±	0.00075	0.36^{a}	±	0.59	F(3,8)=0.736, p=0.560
Sb	1.49^{a}	±	0.14	2.384^{b}	±	0.033	3.336^{c}	±	0.052	3.139^{c}	±	0.051	F(3,8)=323.140, p<0.0001
Cs	0.00278^{a}	±	0.00018	0.00228^{a}	±	0.00013	0.00402^{b}	±	0.0003	0.003461^{c}	±	0.000096	F(3,8)=46.550, p<0.0001
Ba	1.923^{a}	±	0.027	3.57^{b}	±	0.1	4.42^{c}	±	0.11	3.81^{b}	±	0.11	F(3,8)=386.508, p<0.0001
Pb	1.28^{a}	±	0.15	2.301^{b}	±	0.078	2.964^{c}	±	0.061	2.528^{b}	±	0.013	F(3,8)=194.467, p<0.0001

*different superscript lower case letters represent statistically significant differences
